# Rylene Dye-Loaded Polymeric Nanoparticles for Photothermal Eradication of Harmful Dinoflagellates, *Akashiwo sanguinea* and *Alexandrium pacificum*

**DOI:** 10.3390/bioengineering9040170

**Published:** 2022-04-11

**Authors:** Naz Fathma Tumpa, Mingyeong Kang, Jiae Yoo, Sunju Kim, Minseok Kwak

**Affiliations:** 1 Department of Chemistry, Pukyong National University, Busan 48513, Korea; nazabsolve2205@gmail.com; 2 Industry 4.0 Convergence Bionics Engineering, New-Senior’ Oriented Smart Health Care Education Center, Pukyong National University, Busan 48513, Korea; mgkang@pukyong.ac.kr; 3 Division of Earth Environmental System Science, Pukyong National University, Busan 48513, Korea; jiae502@gmail.com; 4 Department of Oceanography, Pukyong National University, Busan 48513, Korea

**Keywords:** rylenes, sIPNs, NIR irradiation, photothermal materials, microalgae, red tides

## Abstract

In the era of climate changes, harmful dinoflagellate outbreaks that produce potent algal toxins, odor, and water discoloration in aquatic environments have been increasingly reported. Thus, various treatments have been attempted for the mitigation and management of harmful blooms. Here, we report engineered nanoparticles that consist of two different types of rylene derivatives encapsulated in polymeric micelles. In addition, to avoid dissociation of the aggregate, the core of micelle was stabilized via semi-interpenetrating network (sIPN) formation. On two types of the marine red-tide dinoflagellates, *Akashiwo sanguinea* and *Alexandrium pacificum*, the nanoparticle uptake followed by fluorescence labeling and photothermal effect was conducted. Firstly, fluorescence microscopy enabled imaging of the dinoflagellates with the ultraviolet chromophore, Lumogen Violet. Lastly, near-infrared (NIR) laser irradiation was exposed on the Lumogen IR788 nanoparticle-treated *Ak. Sanguinea.* The irradiation resulted in reduced cell survival due to the photothermal effect in microalgae. The results suggested that the nanoparticle, IR788-sIPN, can be applied for potential red-tide algal elimination.

## 1. Introduction

Polymeric micellar aggregates have been promising nano-carriers for tumor imaging and drug delivery applications [1,2,3]. These polymeric nanoparticles attracted much attention because of their controllable physicochemical properties such as size, surface charge, degradability, and functionalization [4,5]. Above their critical micelle temperature (CMT) and concentration (CMC), the block copolymers can self-assemble into supramolecular structures composed of a hydrophobic core for loading hydrophobic molecules and a hydrophilic shell that can protect from dissociation in the aqueous environment [6,7,8,9].

Among those, a Pluronic^®^ block copolymer consisting of poly(ethylene oxide-*b*-propylene oxide-*b*-ethylene oxide) (PEO-*b*-PPO-*b*-PEO) has been widely studied as a therapeutic-agent carrier because of its excellent bioavailability [10,11,12]. In aqueous media above its CMT and CMC, Pluronic^®^ can spontaneously form nano-sized core-shell micellar structures having a hydrophobic core composed of PPO segments and a shell dominated by PEO segments. The PEO chain of Pluronic^®^ can form hydrogen bonds with aqueous molecules resulting in a tight shell around the micellar core, which prevents the clearance of hydrophobic molecules from the core [13,14,15,16].

Although Pluronic^®^ has been considered to be a promising drug carrier, these micelles, which have soft cores, are thermodynamically less stable than other polymeric micelles with a solid core such as poly(styrene)-*b*-poly(ethylene oxide). To stabilize Pluronic^®^ micelle, many stabilization methods have been conducted by other groups focused on PEO and PPO functionalization [17,18,19,20]. Mostly, reported methods not only require complicated organic synthesis routes but also influence the micellization properties of Pluronic^®^ molecules [17]. The innovative method to stabilize Pluronic^®^ micelle has been developed by Petrov et al. via the formation of sIPN in the core of Pluronic F-68 using pentaerythritol tetraacrylate (PETA) as a crosslinker [21]. The interpenetrating networks of poly(PETA) and polyether chains were stabilized Pluronic^®^ micelle from degradation below its CMC or CMT. Thus, the resulting sIPN core firmly holds small molecules within the network.

Among small molecules trapped in the core, the loading of hydrophobic dyes has been widely done using polymeric micelles [22]. In particular, rylene dyes are polyaromatic chromophores with exceptional photophysical properties such as high photostability and intense absorption [23]. Rylene dyes with multiple naphthalene units are known to absorb longer wavelengths up to 1000 nm [24].

Harmful algal blooms (HABs) by cyanobacteria and dinoflagellates in aquatic environments have globally increased in intensity and frequency during the past decade [25,26,27]. HABs cause mass mortalities of farmed fish, leading to severe economic losses [28,29]. Moreover, toxigenic HAB species produce a range of potent algal toxins that impact human health, such as a paralytic toxin, neurotoxin, and amnestic shellfish poison [30]. To mitigate and manage the HABs, several treatments including filtration, flocculation, and degradation are applied in the field [31,32]. However, the currently used physical, chemical, biological, and converged control can achieve temporary and partial effect, but are not sustainable for a long period. Therefore, research on new methods for effective algae removal is needed. A method that has high algae removal efficiency and does not cause secondary pollution is required. In addition, it is necessary to consider situations such as large-scale aquatic environments with large changes in seasonal and hydraulic environmental factors.

**Figure 1:**
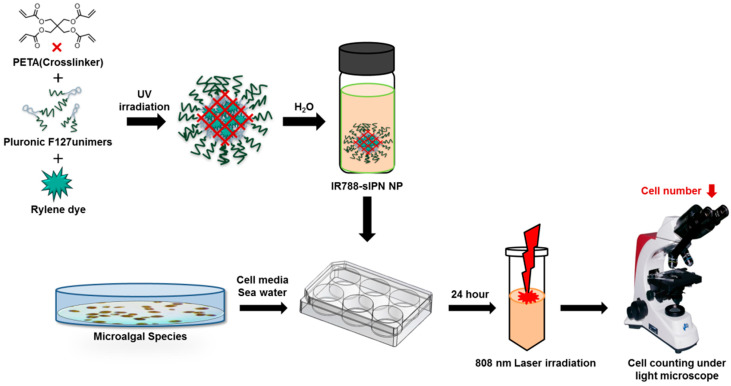
Schematic diagram of rylene-dye-loaded NPs uptake to algal cell experiment.

As illustrated in Figure 1, polymeric nanoparticles (NPs) were loaded with rylene dyes and stabilized to avoid dissociation of the polymeric micelle. Pluronic^®^ F127, a triblock copolymer consisting of hydrophobic PPO chain and hydrophilic PEO chain, was used as a base for the polymeric micelle with Lumogen^®^ IR788 (IR788) and Lumogen^®^ violet (LV) as loaded moiety [33,34,35,36]. The rylene dye-loaded NP was further stabilized via sIPN within the core of the micelle using a PETA. UV irradiation was used to promote the network formation of crosslinker, holding the PPO core within micelle [37,38]. The effects of LV-sIPN NP and IR788-sIPN NP on two dinoflagellate species such as *Ak. Sanguinea* and *A. pacificum* were evaluated after feeding NP in the algal media, and later *Ak. sanguinea* species was selected for NIR (808 nm) laser irradiation treatment under different exposure conditions.

In addition, most of the previous microalgae studies regarding nanoparticles evaluated the toxicity of NPs themselves, but there has been very little study evaluating the effects to microalgae under IR exposure where the majority of energy is transferred from sunlight in the aquatic environment. Algal media without NP were used as control compared to media containing NP. Two red-tide species were studied as the model species owing to their importance as a primary harmful algal bloom species in the aquatic environment.

## 2. Materials and Methods

### 2.1. Materials

#### 2.1.1. Reagents and Solvents for NP

Lumogen violet and IR788 were purchased from BASF (Ludwigshafen, Germany). Pluronic^®^ F127 (MW 12,600) and PETA were purchased from Sigma Aldrich (Burlington, MA, USA). Chloroform (99.8%, HPLC grade) was purchased from Samchun (Seoul, Korea), and acetone (99.9%) was purchased from DUKSAN (Ansan, Korea). Ultra-pure water (18 MΩ) purified on a MilliQ-Millipore system (Merck Millipore, Burlington, MA, USA) that was used throughout the synthesis of NP.

#### 2.1.2. Cell Isolation and Cultures

Two species of dinoflagellates, *Ak. sanguinea* (Strain AS-USA) and *A. pacificum* (Strain AP-LOHABE04), were established in clonal cultures using seawater samples collected during blooms from the Chesapeake Bay (USA) in 2015 and from Masan (Korea) Bay in June 2016, respectively. Cells were individually isolated by a capillary pipette, washed five times in sterile seawater, and transferred to polystyrene cell culture plates containing sterile f/2-Si medium under an inverted microscope. Cells were grown in sterile f/2-Si medium with a salinity of 27.5 for *Ak. sanguinea* and 30 for *A. pacificum* at 20 °C on a 14:10 light-to-dark cycle of cool-white fluorescent lamps of 80 μmol photons m^−2^ s^−1^.

### 2.2. Methods

#### 2.2.1. Preparation of Dye Loaded sIPN

To prepare the PETA film, 125 μL of 100 mg/mL PETA stock solution dissolved in chloroform was placed in an empty vial with a micropipette and dried in a fume hood for 24 h at ambient condition. F127 stock solution dissolved in chloroform of 500 mg F127 and Lumogen dye (Lumogen^®^ violet for 0.002%, Lumogen^®^ IR788 for 3% ratio based on F127) were mixed in the other vial. Solvent, chloroform, was evaporated using a rotary evaporator under reduced pressure so that the F127 LV film was prepared. To prepare a 10 wt% F127 solution above CMC [39], the film was hydrated with 4.5 mL ultrapure water. The solution was transferred to a PETA film prepared in advance and agitated at room temperature overnight using an orbital shaker at 200 rpm. The inside of the vial was filled with argon gas. The solution was irradiated using OmniCure series 2000 (Lumen Dynamics, Mississauga, ON, Canada) at 1.5 W/cm^2^ for 10 min at 50 °C to promote crosslinking of PETA in the micelle core. Dye-loaded sIPN was filtered using 0.2 μm syringe filter (Minisart^®^ Sartorius Stedium, Göttingen, Germany) to remove residual impurities and stored in the clean 20 mL glass vial for further characterization.

#### 2.2.2. CMT Test

The CMT test was done by incubating of sIPN NP at a temperature below its CMT overnight then followed by centrifugation at 4 °C for 10 min at 14,000 rpm using R17 Micro Refrigerated Centrifuge (Hanil Science Industrial, Gimpo, Korea). The supernatant was transferred into a clean tube for further characterization. CMT test was done to evaluate the sIPN formation in the core of micelle. The values of CMT vary depending on temperature and concentration of amphiphilic polymers, and the CMT for a given concentration of F127 in H_2_O (10 wt%) was 17 °C [40]. Both LV-sIPN NP and IR788-sIPN NP were incubated overnight at 4 °C, a temperature way below CMT. Absorption spectra of the samples were recorded using SpectraMax M2 (Molecular Devices, San Jose, CA, USA) in a quartz cuvette with a 10 × 10 mm light path (Hellma Analytics, Müllheim, Germany).

#### 2.2.3. Dynamic Light Scattering

To confirm the size distribution of sIPN NPs, Zetasizer Nano ZS90 (Malvern Instruments, Worcestershire, UK) was used. The particle size was measured at 25 °C. The NP solution was diluted to 20 times with water and filtered using a 0.2 μm syringe filter to remove unwanted aggregates. The data are average of three times measurement.

#### 2.2.4. Settling Assay of NP Uptake by Algal Species

We applied the two dinoflagellate species *Ak. Sanguinea* and *A. pacificum* in exponential growth phases for the NP uptake experiments. Sterilization of algal culture media was done with Spirit-lamp. Stock cultures of the two species were diluted using a fresh f/2 medium and distributed to the 6-well plates (SPL Life Sciences Co., Pocheon, Korea) to achieve triplicate treatments and controls with initial cell concentrations of 2500 cell mL^−1^. A 2 μL of LV-sIPN NP was added into triplicate treatments containing each species. The NP and algae solutions were carried out using Lab Companion SKF 2050 Shaker (Jeio Tech, Daejeon, Korea). All experiments were incubated in the dark condition at 20 °C. For determination of cell abundances and uptake rates of LV-sIPN NP, 1 mL subsamples were taken at 0 h and 24 h and fixed with glutaraldehyde (final concentration of 1% *v*/*v*). Cell abundance and the proportion of cells containing NP were estimated by scanning triplicate Sedgewick Rafter counting chambers (SR chamber) at ×100 magnification under a Zeiss Axio Imager2 microscope (Carl Zeiss Inc., Oberkochen, Germany) with epifluorescence illumination.

#### 2.2.5. Statistical Analysis

All statistical analysis data herein are represented as the mean ± standard error of the mean. The statistical significance of differences among each experimental group was calculated using analysis of variance, and either a Bonferroni posttest or an unpaired Student’s *t*-test. All *p*-values less than 0.05 were considered significant statistically.

#### 2.2.6. SEM Imaging

A 15 mL culture of sIPN NP retained dinoflagellate cells was centrifuged at 3300× *g* for 1 min and discarded the supernatant. The cells were fixed with glutaraldehyde to achieve a final concentration of 2% and stored at 4 °C for 1 h. The fixed specimen was filtered on 3 μm pore-sized membrane filters and then washed in distilled water for 1 h. After that, the sample was dehydrated in ethanol of 25, 50, 70, 80, 99, and 100% gradient for 12 min at each step. Dehydrated *A. pacificum* was a critical point dried with liquid CO_2_ by Leica EM CPD300 and *Ak. sanguinea* was dried by hexamethyldisilazane. Then cells were coated with gold for 8 min and examined by CX-200 (COXEM Inc., Daejeon, Korea) scanning electron microscopy (SEM) for imaging.

#### 2.2.7. NIR Laser Irradiation—Power Variation

An irradiation experiment was conducted irradiating PSU-H-LED 808 nm laser source/machine (Changchun New Industries, Changchun, China) on algae cells. Six well-plate set up after initial counting and counting within time all were the same as the methods used for earlier sets of experiments done in settling assay. *Ak. sanguinea* was treated with LV-sIPN NP for uptake confirmation and IR788-sIPN NP for photothermal material loading. Both LV-sIPN NP and IR788-sIPN NP were treated at a ratio of 1:4000. Irradiation applying times were counted using stopwatch. Different laser power was performed for the treatment of NP uptake algae cells with a fixed solution. The laser power was adjusted using a radiometer (R2000 from Omnicure, Excelitas Technologies Corp., Waltham, MA, USA). LV-sIPN NP and IR788-sIPN NP were used while the power was 1.5 W/cm^2^, again only IR788-sIPN NP was applied for the treatment of NP uptake algae cells while varying the power of 1.0 and 0.5 W/cm^2^.

#### 2.2.8. NIR Laser Irradiation—Time Variation

LV-sIPN NP and IR788-sIPN NP uptake algae samples were irradiated using an 808 nm laser when power was 1.5 W/cm^2^ and again only IR788-sIPN NP uptake algae samples were irradiated using an 808 nm laser with 1.0 and 0.5 W/cm^2^ power. Irradiation was done inside a makeshift cold chamber, and irradiation times were set depending on power for 1.5 W/cm^2^ irradiation times were 15, 20, and 30 min, for both 1.0 W/cm^2^ and 0.5 W/cm^2^ power irradiation times were 10, 30, 45, and 60 min.

## 3. Results and Discussion

To apply the hydrophobic fluorescent dye and photothermal material to red tides, stable polymeric NP was prepared using F127 as described above. To confirm the stability of the prepared dye-loaded sIPN NP, absorbance and fluorescence spectra under the temperature condition under CMT were compared with the spectra at room temperature in water.

**Figure 2:**
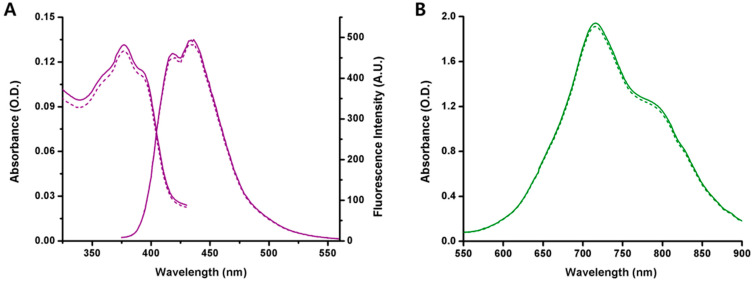
(**A**) Absorption and fluorescence spectra of 0.002% LV-sIPN NPs and (**B**) absorption spectra of 3% IR788-sIPN NPs before and after CMT test.

Figure 2 shows absorption and fluorescence spectra for LV-sIPN NP and absorption spectra for IR788-sIPN NP, which had less than 5% difference before and after CMT test. As specified in Table S1, after CMT test, the obtained result revealed that both F127 sIPN NPs can maintain their cores at a temperature below the CMT.

After CMT test, though, it became apparent that the optical density (O.D.) value of IR788 micelle drastically decreased up to 91% (Table S1) because of the dissociation of micelle into unimers under CMT, releasing most of the loaded dyes in the process. This release of dye was not observed in IR788-sIPN NP, as proven by an O.D. decrease of only 0.6%, owing to the structurally fixed core of the sIPN.

The hydrodynamic diameters of LV-sIPN NP and IR788-sIPN NP determined by DLS were 20.21 and 25.58 nm, respectively. These results are similar to 22 nm, which calculated values through molecular dynamics simulation [41]. Figure S1 shows the size distribution of LV-sIPN NP, LV-micelle NP, IR788-sIPN NP, and IR788-micelle NP. The particle size of IR788-sIPN NP was larger than that of LV-sIPN NP because quaterrylene IR788 has a 4.8 times larger chemical structure, causing it to form a somewhat larger aggregate. The size of sIPN NPs reported in the previous study was 7 nm [37]. Because the dense core is clearly observed under TEM conditions, the observed size is smaller than the hydrodynamic volume determined by DLS measurements.

**Figure 3:**
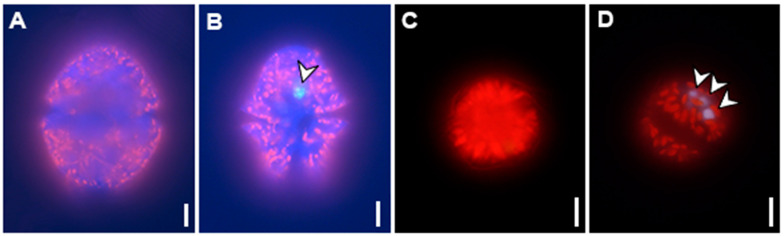
Fluorescence microscopy images of dinoflagellates non-feeding and feeding on LV-sIPN NPs. (**A**,**B**) *Akashiwo sanguinea*, (**C**,**D**) *Alexandrium pacificum*. (**A**,**C**) Cells with no NP, (**B**,**D**) Cells containing LV-sIPN NPs. The self-fluorescence of the cell is red and the LV-sIPN NPs are blue (arrow). Scale bars = 10 μm.

Retention of sIPN NP by red-tide algal cells was observed and investigated to determine which dinoflagellate species has higher NP uptake ability. LV-sIPN NP was used as an ideal material for uptake experiment due to easy detection under an epifluorescence microscope as shown in Figure S2. When fluorescence of LV-sIPN NP was observed at the location of the cell identified by the microscope, it was considered that it had taken up NP. To check the uptake pattern of dinoflagellate, 24 h after the NP treatment, the total cell numbers and the number of cells containing NP were counted and compared with the number of cells at 0 h. Figure 3 shows two dinoflagellate species images. Both the dinoflagellate species retained LV-sIPN NP and were clearly distinguished from normal cells in the fluorescence images.

**Figure 4:**
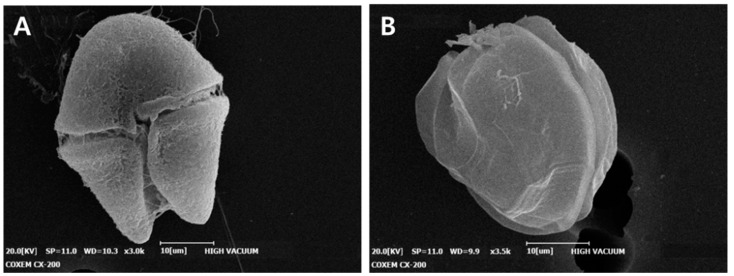
SEM images of LV-sIPN treated algae (**A**) *Akashiwo sanguinea*, (**B**) *Alexandrium pacificum*.

NP used in various fields is a potential hazard that can affect aquatic ecosystems due to their properties such as size and chemical composition. The adsorption or uptake of NP to algae can inhibit photosynthetic processes or cause physical membrane damage [42]. Figure 4 shows the SEM imaging results of dinoflagellate cells treated with sIPN NP. We found that there is no damage to the surface of cells treated with sIPN NP. LV-sIPN-treated algae cells have no difference compared to SEM images of normal cells that have not been treated [43,44].

**Figure 5:**
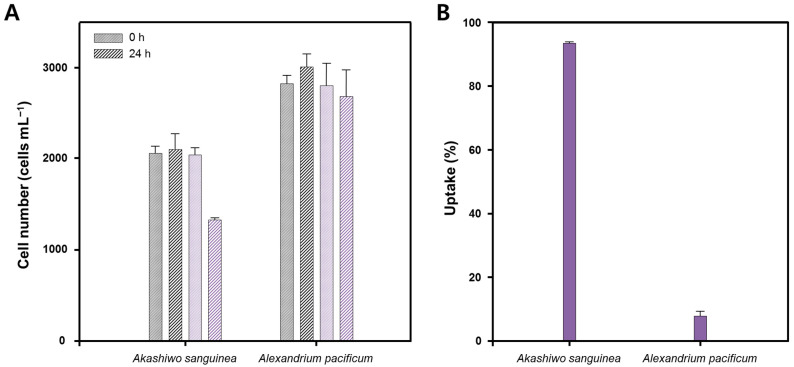
Monitoring the uptake for dinoflagellate species, *Akashiwo sanguinea* and *Alexandrium pacificum*, after 24 h of LV-sIPN NPs treatment. (**A**) Effect of LV-sIPN NPs on cell growth. It shows the change in the number of cells immediately after nanoparticle treatment (purple) and after 24 h (hatched purple). As a control, the number of untreated red-tide cells (grey) and the cell number after 24 h of incubation (hatched grey) were also observed. (**B**) Comparison of LV-sIPN NP uptake rate of dinoflagellate species after 24 h.

Figure 5A represents the mean cell number of *Ak. sanguinea* and *A. pacificum* during treatment with LV-sIPN NP at 0 h and 24 h under light-to-dark conditions. After 24 h of NP treatment to *Ak. sanguinea*, the number of cells retained by 64%. The cells containing LV-sIPN NP among living cells were 95% of the total as shown in Figure 5B. While *A. pacificum* retained by 94% after LV-sIPN NP treatment, it was observed that only 7.93% of cells had uptake of LV-sIPN NP. *Ak. sanguinea* showed higher uptake ability compared to *A. pacificum* cells due to ornamented theca on the cell outer layer. We chose *Ak. sanguinea* for further laser treatment investigation due to higher NP uptake ability.

**Figure 6:**
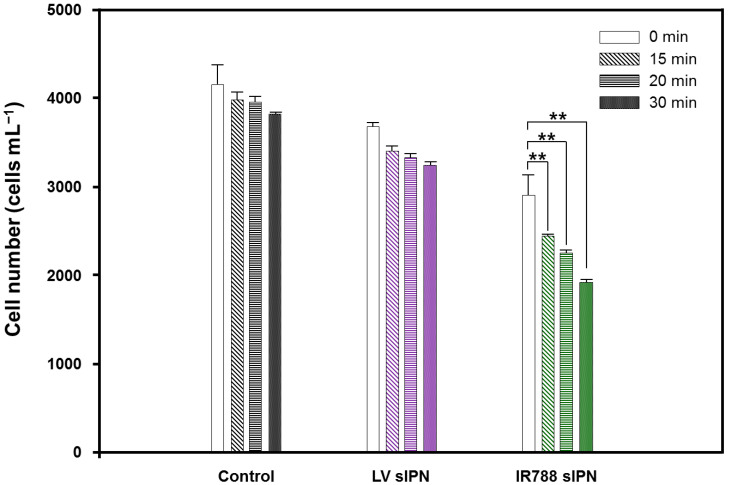
Effect of laser irradiation (808 nm) under 1.5 W/cm² on *Akashiwo sanguinea* treated with LV- and IR788-sIPN NPs at different irradiation time. One-way ANOVA, mean  ±  SEM, ** *p* < 0.01.

LV-sIPN and IR788-sIPN NP were applied on *Ak. sanguinea* and irradiated for 15, 20, and 30 min by 808 nm laser under 1.5 W/cm^2^ power. The IR788-sIPN NPs solution could reach hyperthermia under 808 nm laser irradiation [45]. In this experiment, the power that can kill cells by generating local heat inside the cells without causing changes in water temperature was selected. Algal cell number decreased with increasing laser-light exposed time. Figure 6 shows decreasing cell number of *Ak. sanguinea* with NP uptake and without NP as control according to the irradiation time of 808 nm laser with 1.5 W/cm^2^. Cell number decreased with increasing irradiation time up to 30 min under the same cell concentration. Significant differences in algal cell numbers were observed for irradiation times in cells with different NP uptake.

After 30 min of laser irradiation, the cells of the control group decreased by 7%. Laser irradiation had no significant effect on the control cell due to NP absence. On the other hand, the cell number of the experimental group that was exposed to LV-sIPN NP decreased by 14% after irradiation for 30 min. IR788-sIPN NP showed the highest number of cells decreased compared to both control and LV-sIPN NP, which was 32% with 30 min of irradiation time. This observation suggests that *Ak. sanguinea* was removed by the material loaded inside the NP. IR788 inside the NP affects microalgae cells as a photothermal material that generates heat by the photoexcitation when it is exposed to NIR wavelength light.

**Figure 7:**
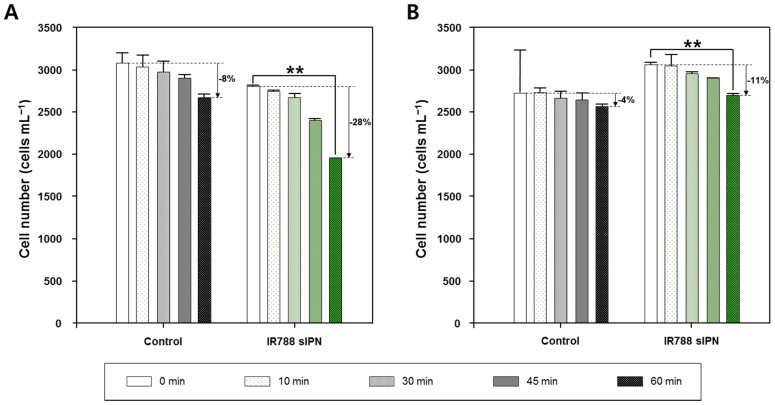
Change of cell number after laser irradiation under (**A**) 1.0 and (**B**) 0.5 W/cm² on *Akashiwo sanguinea* at different irradiation time. Noted decrease percentage is relative to each non-irradiated control (0 min). One-way ANOVA, mean  ±  SEM, ** *p* < 0.01.

Next, to determine the effect of accumulated light energy on *Ak. sanguinea*, IR788-sIPN NP-treated *Ak. sanguinea* cells were irradiated for 10, 30, 45, and 60 min at 808 nm laser with different power, under 1.0 and 0.5 W/cm^2^. In all cases, algal cell numbers gradually decreased with increasing laser exposed time. Figure 7 shows the effect of laser irradiation on both NP-treated cells and control including cell decrease percentage at different irradiation times. The highest number of cells decreased for the control plate was 8% and 4% for 1.0 and 0.5 W/cm^2^ with 60 min of irradiation time respectively. In contrast, the experimental group treated with IR788-sIPN NP uptake showed that the number of cells decreased up to 28% and 11%. This trend in cell decrease can be significantly seen after 45 min of irradiation. 

## 4. Conclusions

LV and IR788-sIPN NP, core-stabilized polymeric micelle loaded with rylene dyes, were successfully prepared via self-assembly followed by sIPN formation. The sIPN structure greatly improved the stability of NP as shown by CMT test. Herein, we assessed the uptake ability and effect of LV-sIPN NP and IR788-sIPN NP on marine harmful microalgae *Ak. sanguinea* and *A. pacificum*. NP uptake by *Ak. sanguinea* was found to be higher compared to that of *A. pacificum*, confirmed by fluorescence image after 24 h light-to-dark conditions. IR788-sIPN NP had an immediate effect on the removal of *Ak. sanguinea* cells at different laser irradiation times and power than LV-sIPN NP due to the photothermal property of IR788. Therefore, we concluded that IR788-sIPN NP is a highly potent material for red-tide algal eradication. Our next goal is to eliminate algal species taking up IR788-sIPN NP by local heat generated under intense sunlight because solar radiation in the infrared range (longer than 760 nm) is about 40%. To achieve this goal in the future, several aspects in the NP need to be considered such as biodegradation, environmental effect, and aquatic food chains.

## Data Availability

The data presented in this study are available on request from the corresponding author.

## Supplementary Materials

The following supporting information can be downloaded at: https://www.mdpi.com/article/10.3390/bioengineering9040170/s1, Figure S1: Particle size distribution of (A) LV-sIPN NP, (B) LV-micelle NP, (C) IR788-sIPN NP and (D) IR788-micelle measured by dynamic light scattering, Figure S2: Fluorescence microscopy images of dinoflagellates feeding on LV-sIPN NPs. (A) Akashiwo sanguinea, (B) Alexandrium pacificum. The self-fluorescence of the cell is red and the LV-sIPN NPs are blue, Table S1: Absorbance of LV-sIPN NP, LV-micelle NP, IR788-sIPN NP, and IR788-micelle before and after CMT test.
