# Supplementary material for: Rylene Dye-Loaded Polymeric Nanoparticles for Photothermal Eradication of Harmful Dinoflagellates, Akashiwo sanguinea and Alexandrium pacificum
Source: Bioengineering (Basel). 2022 Apr 11;9(4):170. doi: 10.3390/bioengineering9040170 (PMC9026783; doi:10.3390/bioengineering9040170)
Supplement: Supplementary file 1 [file bioengineering-09-00170-s001.zip › bioengineering-1638271-SI.pdf]

Supporting Information

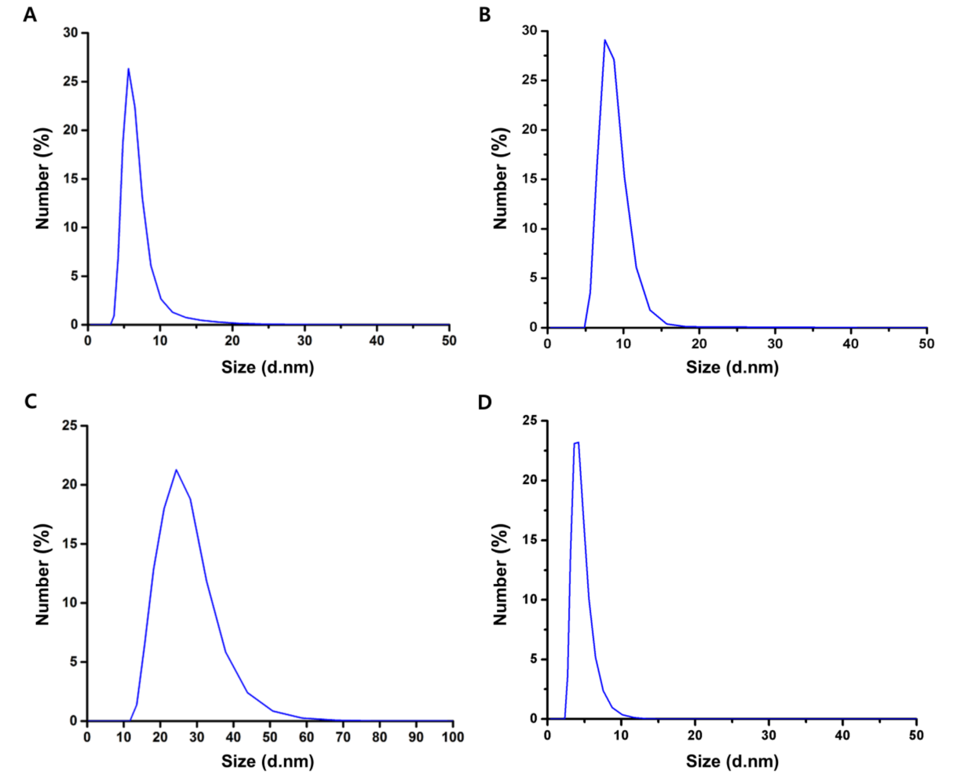

Figure S1. Particle size distribution of (A) LV-sIPN NP, (B) LV-micelle NP, (C) IR788-sIPN NP and (D) IR788-micelle measured by dynamic light scattering.

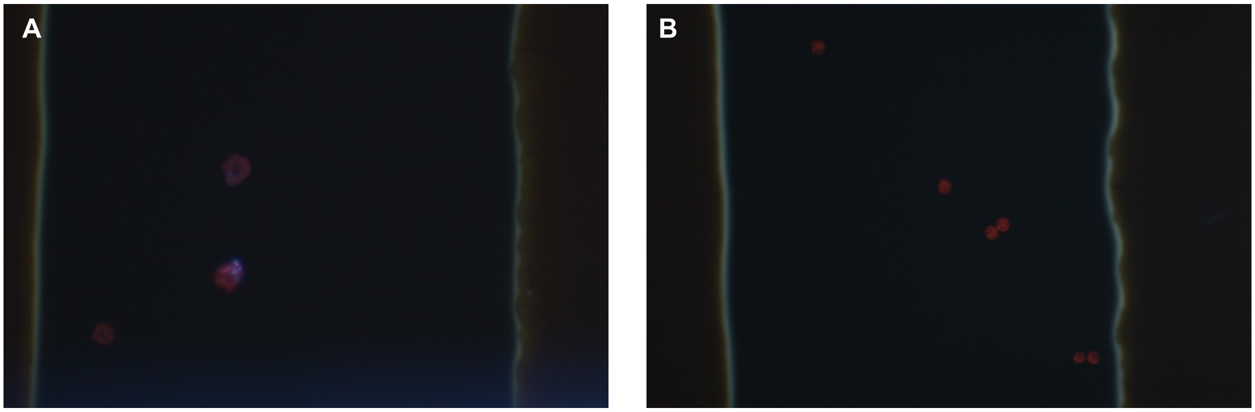

Figure S2. Fluorescence microscopy images of dinoflagellates feeding on LV-sIPN NPs. (A) *Akashiwo sanguinea*, (B) *Alexandrium pacificum*. The self-fluorescence of the cell is red and the LV-sIPN NPs are blue.

Table S1. Absorbance of LV-sIPN NP, LV-micelle NP, IR788-sIPN NP and IR788-micelle NP samples before and after CMT test.

Sample	Absorbance[a] of sample stored at		Ratio intensity (4°C/ 25°C)
	25°C (RT)	4°C (CMT)	
0.002% LV sIPN NP	0.5569	0.5454	0.979
0.002% LV micelle NP	0.1620	0.1299	0.802
3% IR788 sIPN NP	2.3330	2.2490	0.964
3% IR788 micelle NP	2.4689	0.2260	0.092

[a] O.D. values at 377 and 716 nm for LV and IR788, respectively.
